# To the Readers

**Published:** 2010

**Authors:** Roya Kelishadi

**Affiliations:** Founding Editor, International Journal of Preventive Medicine

By definition, Medicine is the art and science of healing. It encompasses a range of health care practices evolved to maintain and restore health by the prevention and treatment of illness (http://en.wikipedia.org/wiki/Medicine). Upon graduation, medical students take the Hippocratic Oath, including one of its quotes saying “*I will prevent disease whenever I can, for prevention is preferable to cure*.”

(http://www.pbs.org/wgbh/nova/doctors/oath_modern.html).

Even though the Medicine’s main goal is to prevent or cure diseases, medical students are mostly trained for diagnosis and treatment of disease. As a result, during their practice as physicians, they do not consider their important role in disease prevention and health promotion. Many physicians agree with Dr. C. Everett Koop who said “*I don’t think a medical student is ever told what his mission in life is. Certainly no one told me when I was a medical student what was expected of me as a lifetime goal in assuming the role of a physician*.” Moreover, many physicians do not consider that many parts of their efforts for disease management are, in fact, secondary or tertiary levels of prevention.

There has been a debate over the relative importance of prevention vs. treatment; obviously they should not be considered as two opposite sides of a health system, which requires both prevention and treatment.

Preventive medicine has a long history; however in some parts of the world, it is functioning as an arm of public health, whereas in other parts physicians practice it with an individualized approach. Our new journal aims to serve as a focal point for debates and exchange of knowledge and experience in the entire field of preventive medicine among researchers in a global context. By considering the primordial, primary, secondary and tertiary levels of prevention, it publishes articles in the area of preventing non-communicable and communicable diseases as well as the promotion of individual and community health. Of particular emphasis are papers that address applied research studies that aim to promote health, to preserve health, to retard the progress of the disease and disability, to restore health by rehabilitation, and to minimize suffering and distress.

As Preventive Medicine covers both population-based and clinical approaches to health care, and considering our journals’ motto: “*Preventive Medicine: From Molecular to Population Studies*”, international organizations and researchers of various field of expertise are contributing to provide an international forum for publication of high quality papers. As noticeable by the research fields of the collaborating organizations and Editorial Board members, we considered the main competencies of Preventive Medicine, i.e. Biostatistics/epidemiology, Management/administration, Clinical Preventive Medicine and Occupational Health/Environmental Health. Hopefully, the papers published in the first issue of our journal can represent, at least in part, the journal’s areas of interest.

We are proud to begin this initiative from a region where distinguished scientists, as Avicenna have been the forerunner of Preventive Medicine; it is our hope that researchers from all over the world would support us to provide a scientific international forum in different fields of Preventive Medicine.

Albert Einstein said:” *Intellectuals solve problems; geniuses prevent them*.”, so let’s try to be genius in our medical care!

